# Integration of Online Omics-Data Resources for Cancer Research

**DOI:** 10.3389/fgene.2020.578345

**Published:** 2020-10-23

**Authors:** Tonmoy Das, Geoffroy Andrieux, Musaddeque Ahmed, Sajib Chakraborty

**Affiliations:** ^1^Molecular Systems Biology Laboratory, Department of Biochemistry and Molecular Biology, University of Dhaka, Dhaka, Bangladesh; ^2^Medical Center – University of Freiburg, Faculty of Medicine, Institute of Medical Bioinformatics and Systems Medicine, University of Freiburg, Freiburg, Germany; ^3^German Cancer Consortium (DKTK), German Cancer Research Center (DKFZ), Partner Site Freiburg, Freiburg, Germany; ^4^Princess Margaret Cancer Centre, University Health Network, Toronto, ON, Canada

**Keywords:** multi-omics, cancer, data-integration, systems biology, proteogenomic analysis

## Abstract

The manifestations of cancerous phenotypes necessitate alterations at different levels of information-flow from genome to proteome. The molecular alterations at different information processing levels serve as the basis for the cancer phenotype to emerge. To understand the underlying mechanisms that drive the acquisition of cancer hallmarks it is required to interrogate cancer cells using multiple levels of information flow represented by different omics – such as genomics, epigenomics, transcriptomics, and proteomics. The advantage of multi-omics data integration comes with a trade-off in the form of an added layer of complexity originating from inherently diverse types of omics-datasets that may pose a challenge to integrate the omics-data in a biologically meaningful manner. The plethora of cancer-specific online omics-data resources, if able to be integrated efficiently and systematically, may facilitate the generation of new biological insights for cancer research. In this review, we provide a comprehensive overview of the online single- and multi-omics resources that are dedicated to cancer. We catalog various online omics-data resources such as The Cancer Genome Atlas (TCGA) along with various TCGA-associated data portals and tools for multi-omics analysis and visualization, the International Cancer Genome Consortium (ICGC), Catalogue of Somatic Mutations in Cancer (COSMIC), The Pathology Atlas, Gene Expression Omnibus (GEO), and PRoteomics IDEntifications (PRIDE). By comparing the strengths and limitations of the respective online resources, we aim to highlight the current biological and technological challenges and possible strategies to overcome these challenges. We outline the available schemes for the integration of the multi-omics dimensions for stratifying cancer patients and biomarker prediction based on the integrated molecular-signatures of cancer. Finally, we propose the multi-omics driven systems-biology approaches to realize the potential of precision onco-medicine as the future of cancer research. We believe this systematic review will encourage scientists and clinicians worldwide to utilize the online resources to explore and integrate the available omics datasets that may provide a window of opportunity to generate new biological insights and contribute to the advancement of the field of cancer research.

## Harnessing Multi-Omics Techniques for Cancer Research

Omics technologies represent high-throughput assays that are designed to identify and quantify all the biomolecules of a particular type –DNA, RNA, protein, and metabolite in a given biological sample. The most popular high-throughput omics techniques include next-generation sequencing (NGS) and mass-spectrometry based techniques. Different omics techniques and their functionalities are listed in Table 1. NGS based techniques are frequently used for genomics, epigenomics, and transcriptomics whereas the mass-spectrometry based techniques are dedicated to proteomics and metabolomics (Chakraborty et al., 2018). NGS based genomics studies are typically designed to analyze the DNA sequence of both coding and non-coding regions in a genome-wide manner. In a more targeted approach, the whole-exome sequencing technique can be used to identify the sequence variation within the exon-sequences of the human genome (Goodwin et al., 2016). NGS based epigenomics techniques include ChIP-seq (chromatin immunoprecipitation), DNase1-seq (DNase I hypersensitive sites – sequencing) or FAIRE-seq (Formaldehyde-Assisted Isolation of Regulatory Elements –sequencing) assay for mapping the DNA-protein interactions or chromatin accessibility, ChiRP-seq (Chromatin Isolation by RNA Purification) for mapping DNA-RNA interaction and whole-genome bisulfite/array-based sequencing for mapping DNA methylation (Furey, 2012). NGS-driven transcriptomics (e.g., RNA-seq) techniques are used for identification and quantification of RNA molecules- including mRNA, miRNA, and other regulatory RNAs in a genome-wide manner (Stark et al., 2019). In contrast to NGS, mass-spectrometry based (LC-MS/MS) techniques are used to identify or quantify the proteins (proteomics) and metabolites (metabolomics) in a high-throughput manner (Blum et al., 2018). Other techniques such as reverse-phase protein arrays (RPPAs) can also be used to quantify different protein molecules using antibodies (Tibes et al., 2006).

**TABLE 1:** The different techniques required for genomics, epigenomics, transcriptomics, proteomics, and metabolomics are given along with their respective functionalities.

Omics type	Technique	Application	References
Genomics	NGS	High throughput whole-genome and whole-exome sequencing	Goodwin et al., 2016
Epigenomics	ChIP-seq	Identification of genome-wide DNA binding sites for transcription factors and associated proteins	Park, 2009
	DNase1-seq	Identification of the active gene regulatory elements across genome	Zhou et al., 2017
	FAIRE-seq	Identification of the DNA regions having regulatory activity	Simon et al., 2012
	ChiRP-seq	Detection of genomic locations of the ncRNAs, such as lncRNAs, and their bound proteins	Chu et al., 2011
	WG bisulfite/array-based sequencing	Determination of the methylation pattern throughout the genome	Furey, 2012
Transcriptomics	RNA-seq	Identifications and quantification of novel transcripts- including mRNA, miRNA, and other regulatory RNAs	Stark et al., 2019
Proteomics	LC-MS/MS based mass-spectrometry	Identification and quantification of proteins abundances on various biological conditions	Blum et al., 2018
	RPPA	Quantification of proteins abundances on various biological conditions	Tibes et al., 2006
Metabolomics	LC-MS based mass-spectrometry	Identifications and quantification of selected molecules involved in metabolic pathways	Raftery, 2014

*NGS, Next-Generation Sequencing; ChIP-seq, Chromatin Immunoprecipitation Sequencing; DNase 1-seq, DNase I hypersensitive sites sequencing; FAIRE, Formaldehyde-Assisted Isolation of Regulatory Elements; ChiRP, Chromatin Isolation by RNA purification; WG, Whole-Genome; RNA-seq, RNA sequencing; LC-MS/MS, Liquid Chromatography Tandem Mass Spectrometry; RPPA, Reverse-phase protein array.*

Alterations at different regulatory layers often lead to the emergence of a cancerous phenotype. Delineation of the underlying mechanisms that drive the acquisition of cancer hallmarks warrants interrogation of the cancer cells from multiple levels. For instance, genomic or epigenomic studies revealed how genetic mutations or epigenetic alterations may drive tumorigenesis (Berdasco and Esteller, 2010; Kandoth et al., 2013). Similarly, transcriptomics and proteomics studies have pinpointed dysregulated genes and proteins in many cancer types (Yanovich et al., 2018; Jiang et al., 2019). However, these single-omics datasets fail to fully untangle the complexity of a disease like cancer. Instead, cancer can be better understood by integrating the multi-omics datasets rather than analyzing single-omics datasets in isolation (Chakraborty et al., 2018; Perez-Riverol et al., 2019).

## Online Resources of Omics-Data

The cancer-associated online omics-data repositories are currently playing a pivotal role in broadening our understanding of cancer-associated cellular processes and mechanisms. It is now possible to integrate different datasets in a systematic way for rapid hypothesis generation and validation. These online resources provide the platform to discover altered molecular patterns in a single cancer type or a pan-cancer manner. Here we provide a comprehensive overview of the online single- and multi-omics resources that are available for different types of cancer.

## Cancer-Specific Multi-Omics Data Resources

In this section, we describe the relative strengths and weaknesses of the cancer-specific omics-data resources along with various analysis and visualization tools across multiple platforms such as genomics, epigenomics, transcriptomics, and proteomics. The comparison of different omics-databases with respect to their data-types and availability of other features such as analysis and visualization portals are depicted in Table 2.

**TABLE 2:** Comparison of online single- and multi-omics databases.

Database name	Database type	Omics-types	Raw-data inspection platform	Clinical data availability	Analysis feature	Multi-cohort/dataset comparison	Multi-omics integration feature	Visualization feature
TCGA	Cancer-specific	Genomics	GDC portal	+	+	+	+	+
		Epigenomics Transcriptomics						
		Proteomics						
ICGC	Cancer-specific	Genomics Transcriptomics	ICGC data portal	+	+	+	+	+
COSMIC	Cancer-specific	Genomics	Available	+	+	+	–	+
TPA*	Cancer-specific	TMA-based IHC*	Available	+	+	+	–	+
GEO	Generalized#	Transcriptomics	Available	+	+	–	–	+
PRIDE	Generalized#	Proteomics	Available	–	–	–	–	–

TCGA, The Cancer Genome Atlas; ICGC, International Cancer Genome Consortium; COSMIC, Catalogue Of Somatic Mutations In Cancer; TPA, The Pathology Atlas; GEO, Gene Expression Omnibus; PRIDE, PRoteomics IDEntifications. *Tissue-microarray based immunohistochemistry; #These databases integrate data from healthy subjects and cancer patients; + indicates availability; – indicates unavailability.

### The Cancer Genome Atlas (TCGA)

**FIGURE 1:**
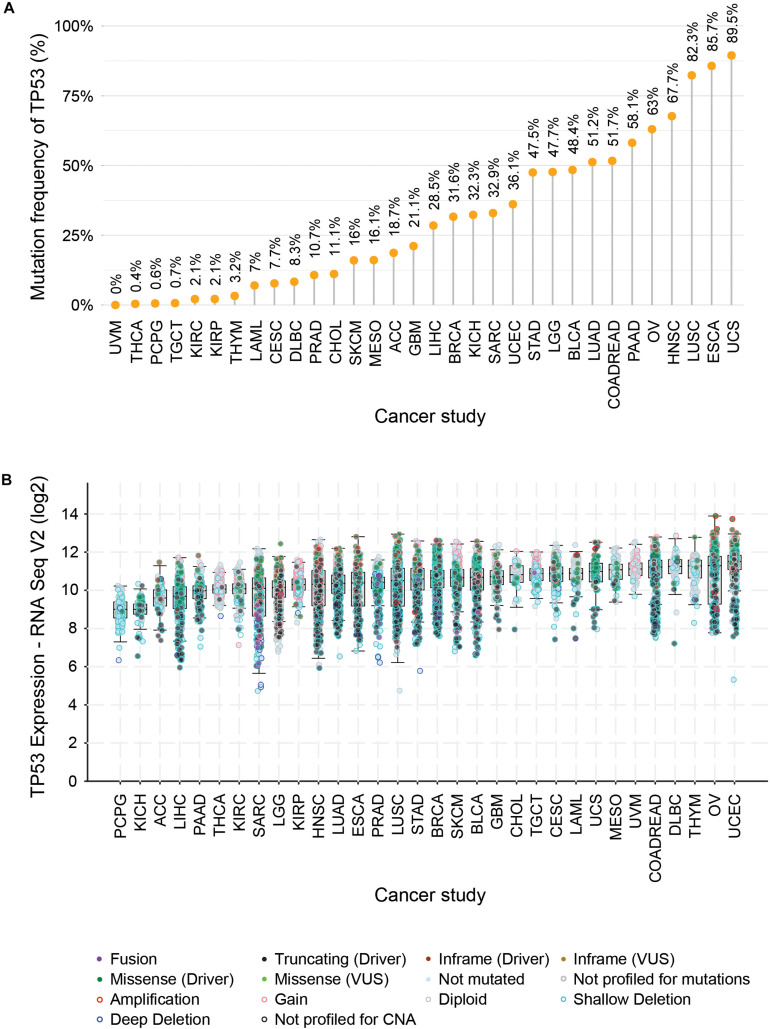
Distribution of mutational frequencies **(A)**, diversities, and mRNA expression profiles **(B)** of *TP53* gene across TCGA cancer types. **(A)** Illustrates the distribution pattern of mutation frequencies of *TP53* – a tumor suppressor gene across 32 TCGA cancer types (ACC, adrenocortical carcinoma; LAML, acute myeloid leukemia; BLCA, bladder urothelial carcinoma; BRCA, breast invasive carcinoma; CESC, cervical squamous cell carcinoma and endocervical adenocarcinoma; CHOL, cholangiocarcinoma; COADREAD, colorectal adenocarcinoma; DLBC, lymphoid neoplasm diffuse large B-cell lymphoma; ESCA, esophageal carcinoma; GBM, glioblastoma multiforme; HNSC, head and neck squamous cell carcinoma; LGG, brain lower grade glioma; LIHC, liver hepatocellular carcinoma; LUAD, lung adenocarcinoma; LUSC, lung squamous cell carcinoma; SKCM, skin cutaneous melanoma; MESO, mesothelioma; OV, ovarian serous cystadenocarcinoma; PCPG, pheochromocytoma and paraganglioma; PAAD, pancreatic adenocarcinoma; SARC, sarcoma; STAD, stomach adenocarcinoma; TGCT, testicular germ cell tumors; THYM, thymoma; THCA, thyroid carcinoma; UCEC, uterine corpus endometrial carcinoma; UCS, uterine carcinosarcoma; UVM, uveal melanoma; KIRC, kidney renal clear cell carcinoma; KICH, kidney chromophobe; KIRP, kidney renal papillary cell carcinoma). **(B)** Depicts the box plot showing the differential mRNA expression of *TP53* where the different mutational events were superimposed and indicated by a different color of solid and hollow circles.

One of the most prominent cancer-specific multi-omics data resources is the Cancer Genome Atlas (TCGA) that has characterized over 20,000 primary cancers and matched normal samples spanning 37 cohorts covering 33 different cancer types (Table 2; Liu et al., 2018). Over the last decade, TCGA managed to generate over 2.5 petabytes of omics data representing genomic, epigenomic, transcriptomic, and proteomic data (Gao et al., 2019). The major molecular characterizations of cancer tissues/cells include NGS based techniques to quantify single nucleotide variants (SNVs), DNA methylation, copy number alterations (CNAs), and mRNA/miRNA expression. Figure 1 represents an example of the mutational frequency of *TP53* – a well-characterized tumor suppressor gene (TSG) across 32 TCGA cancer types. An example of the utility of TCGA multi-omics data integration has been shown by Guo et al. where the authors combined RNA-seq and SNP-array data to identify risk-modulating ncRNA for prostate cancer (Guo et al., 2016). In addition to genome, epigenome, and transcriptome, TCGA attempted to analyze the proteome via the Reverse Phase Protein Arrays (RPPA) technique where antibodies were utilized to measure the expression of around 200 different proteins in individuals with different cancer types (Weinstein et al., 2013). The data generated from RPPA studies have been deposited in “The Cancer Proteome Atlas (TCPA)” (Li et al., 2017). However, non-specificity of the antibodies and low-throughput are the major limitations of RPPA compared to mass-spectrometry based techniques (Chen et al., 2019). To overcome this barrier, TCGA samples from different cancer types were subjected to LC-MS/MS-based mass-spectrometry analysis resulting in the identification and quantification of thousands of proteins. The mass-spectrometry based proteomics data is hosted by the Clinical Proteomic Tumor Analysis Consortium (CPTAC) which strives to broaden the understanding of the tumorigenesis by integrating proteomics data with genomics data (Edwards et al., 2015). Currently, CPTAC hosts proteome, phosphoproteome, and glycoproteome data presenting several types of cancer tissues from TCGA. The integration of different types of omics data generated from TCGA samples involving both NGS and mass-spectrometry techniques has emerged as the multi-omics platform connecting genomics to proteomics enabling the advancement of a new field called proteogenomics (Zhang et al., 2014, 2016; Mertins et al., 2016).

In TCGA, the highest number of samples were collected for the glioma cohort (GBMLGG, *N* = 1129) followed by the breast invasive carcinoma cohort (BRCA, *N* = 1098) and pan-kidney cohort (KIPAN, *N* = 941) (Table 2). The lowest number of samples were obtained for cholangiocarcinoma (CHOL, *N* = 45), lymphoid neoplasm diffuse large B-cell lymphoma (DLBC, *N* = 48), and uterine carcinosarcoma (UCS, *N* = 57). Unlike NGS based studies, mass-spectrometry based proteomics studies have only been performed on eight cancer types (breast invasive carcinoma, colon adenocarcinoma, glioblastoma, renal clear cell carcinoma, lung adenocarcinoma, ovarian serous cystadenocarcinoma, rectum adenocarcinoma, and uterine corpus endometrial carcinoma) (Table 3). However, the benefits of the integration of proteomics with genomics and transcriptomics have already proven useful to generate important and unique biological insights that were not possible with single omics data analysis (Zhang et al., 2014; Mertins et al., 2016). For instance, integration of multi-omics levels was used to uncover the novel altered molecular features and possible therapeutic/prognostic targets in lung adenocarcinoma (Gillette et al., 2020), ovarian cancer (Zhang et al., 2016), breast cancer (Huang et al., 2017) and colorectal cancer (Zhang et al., 2014). In addition to proteomics, phosphoproteomics studies were also conducted simultaneously on the same samples except for rectum adenocarcinoma whereas glycoproteomics was only performed for ovarian serous cystadenocarcinoma (OV) (Table 3). The proteogenomic approach has shown much promise to reveal the latent effect of genomic and epigenomic alterations on the mRNA and protein levels which subsequently led to the acquisition of cancer hallmarks. For example, in colorectal cancer, the proteogenomic approach elucidated the consequence of chromosomal region 20q amplification which was not only restricted to altered mRNA levels but also reflected on the protein levels. The altered protein abundances of Hepatocyte-nuclear factor 4 alpha (HNF4A), Translocase of outer mitochondrial membrane (TOMM34), and SRC proto-oncogene and non-receptor tyrosine kinase (SRC) were directly caused by the 20q amplification event and are likely to play a crucial role in the acquisition of sustained proliferation (Zhang et al., 2014). By considering the translational potential of the proteogenomic studies, the integration of multi-omics data can be an effective strategy to combat cancer by identifying precise diagnostic or prognostic biomarkers and therapeutic targets.

**TABLE 3:** Multi-omics datasets across different TCGA cohorts.

Cohort	Clinical	SNV	CNV	Methylation	mRNA-seq	miRSeq	RPPA	Proteome	Phospho-proteome	Glyco-proteome
ACC	92	92	92	80	80	80	46			
BLCA	412	412	412	412	412	409	344			
BRCA	1097	1044	1098	1095	1097	1078	887	233	233	
CESC	307	305	302	307	307	307	173			
CHOL	45	51	36	36	36	36	30			
COAD	458	433	460	458	459	406	360	164	101	
COADREAD	629					549	491			
DLBC	48	37	50	48	48	47	33			
ESCA	185	184	185	185	184	184	126			
GBM	595	396	599	423	166	0	238	100	100	
GBMLGG	1110					512	668			
HNSC	528	510	526	528	528	523	212			
KICH	113	66	66	66	66	66	63			
KIPAN	941					873	756			
KIRC	537	339	534	533	534	516	478	110	110	
KIRP	291	288	290	291	291	291	215			
LAML	200	149	200	140	188	188	0			
LGG	515	513	515	516	516	512	430			
LIHC	377	375	376	377	376	372	63			
LUAD	522	569	518	579	519	513	365	111	111	
LUSC	504	497	504	503	504	478	328			
MESO	87	83	87	87	87	87	63			
OV	591	443	597	602	492	453	426	283	162	122
PAAD	185	183	185	184	178	178	123			
PCPG	179	179	179	179	179	179	80			
PRAD	499	498	498	498	498	494	352			
READ	171	158	167	165	167	143	131	30		
SARC	261	255	261	261	261	259	223			
SKCM	470	470	470	470	469	448	353			
STAD	443	441	443	443	439	436	357			
STES	628					620	483			
TGCT	134	150	150	150	150	150	118			
THCA	503	496	505	507	507	502	222			
THYM	124	123	124	124	124	124	90			
UCEC	548	542	558	559	559	538	440	104	104	
UCS	57	57	57	57	57	56	48			
UVM	80	80	80	80	80	80	12			

**Total**	**11196**	**10418**	**11124**	**10943**	**10558**	**10156**	**7429**	**1135**	**921**	**122**

**FIGURE 2:**
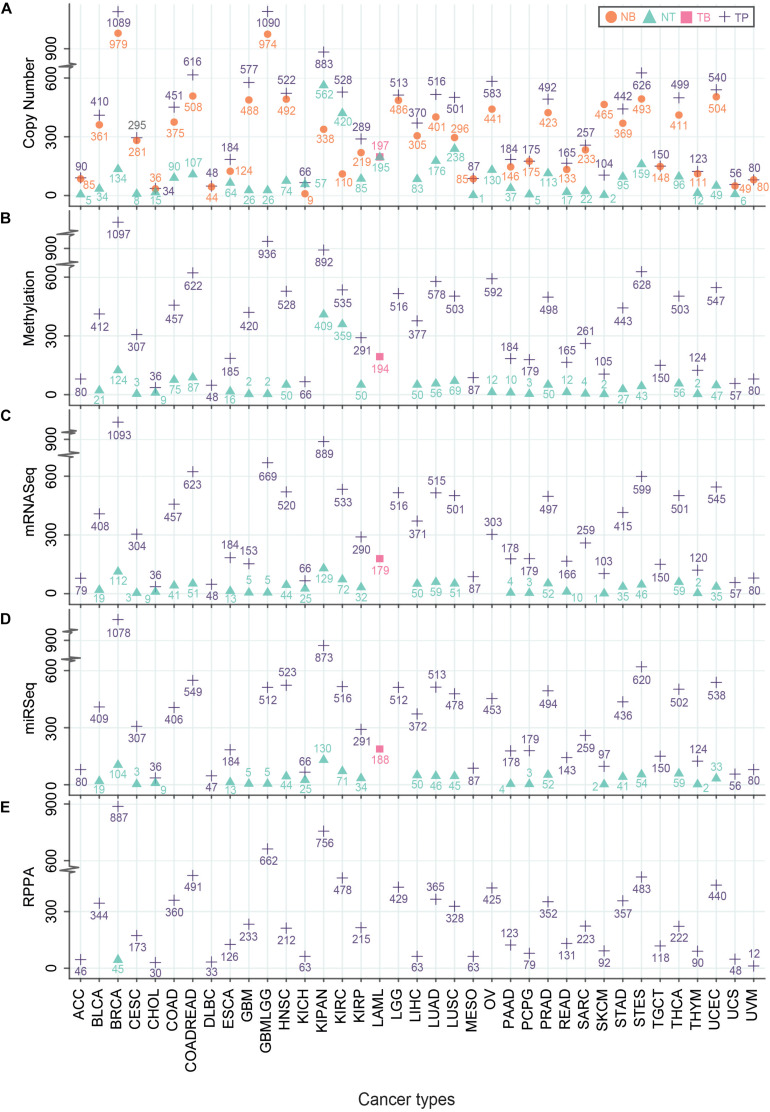
Number of different TCGA-samples across multi-omics platforms. Different types of TCGA samples – Primary Solid Tumor (TP), Blood-Derived Normal (NB), Primary Blood-Derived Cancer – Peripheral Blood (TB) and Solid Tissue Normal (NT) that were used to generate multi-omics data – Genomics: Copy number analysis **(A)**, Epigenomics: Methylation values **(B)**, Transcriptomics: RNA-seq **(C)**, miRNA-seq **(D)** and Proteomics: RPPA **(E)** were plotted. The *Y*-axis represents the number of samples where the *X*-axis indicates TCGA cohorts. Different symbols represent the type of samples.

Understanding the mechanisms of carcinogenesis requires a rigorous comparison of the tumor multi-omics data with normal controls. Although, the expression profiles of non-tumor adjacent tissue (NT) samples vary significantly from the tissues of healthy subjects with normal histological features (Aran et al., 2017), the usage of patient-matched NTs as controls have offered certain advantages. For instance, it minimizes the inter-individual genomic variability (Aran et al., 2017). However, the number of NT samples is not as high as tumor samples (TP) in TCGA. In the case of glioma (GBMLGG), only 51, 5, and 2 non-tumor (NT) samples were used for CNV, RNA abundance, and methylation analyses, respectively (Figure 2). The highest number of NT samples for CNV analysis was obtained for the pan-kidney cohort (KIPAN) (*N* = 562) where the lowest number of NT samples (*N* = 1) was acquired for mesothelioma (Figure 2). The reduced number of patient-matched NT samples may hinder sufficient prospect of harnessing the statistical power to identify the cancer-specific molecular alterations. Nevertheless, TCGA provides the largest repository of cancer-specific multi-omics datasets which offer opportunities for the scientific community to generate novel insights in cancer research.

## Analyses and Visualization Portals of TCGA Data

**FIGURE 3:**
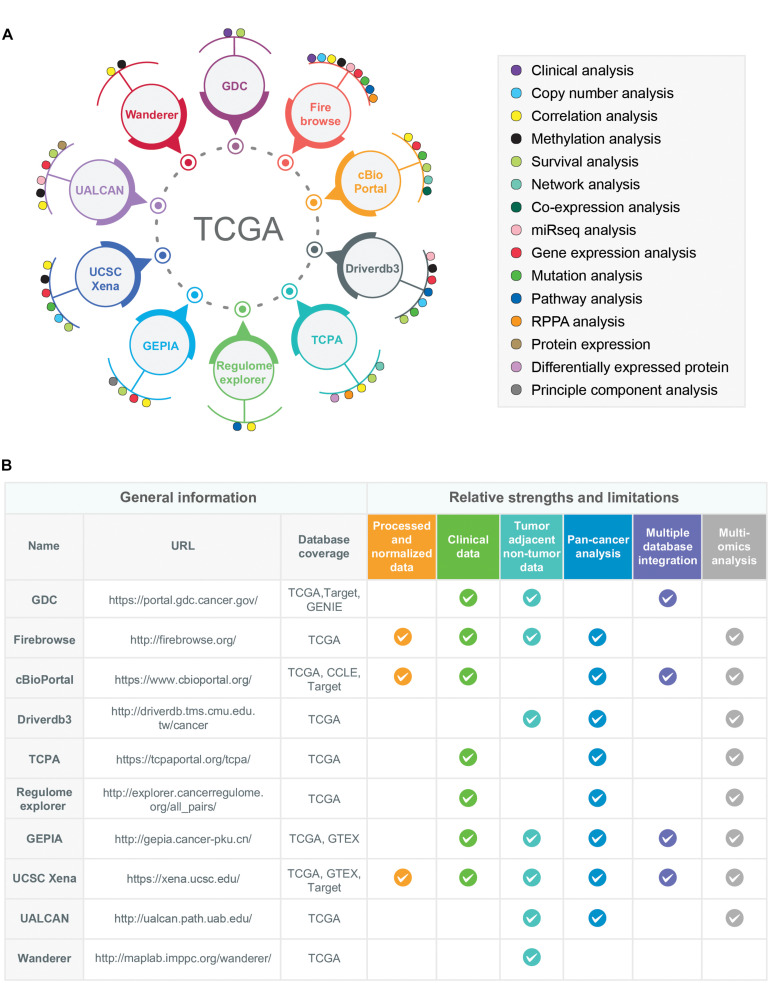
Examples of multidimensional online data portals and visualization tools of TCGA data resources. **(A)** 10 different online data portals, analysis, and visualization tools of TCGA data are shown. The data-analysis dimensions for each of the online data-portals and tools are indicated by colored circles. **(B)** The relative strengths and limitations of TCGA-associated online data portals and tools are shown. For the comparison purposes, six different criteria have been selected: Availability of processed/normalized data, clinical data, tumor-adjacent non-tumor data, pan-cancer analysis features, multiple database integration options (databases other than TCGA), and multi-omics analysis options. The availability of these features in a particular data portals or tools is shown as a tick mark. Moreover, the databases covered by each web portal and tools were also given in the column names “Database coverage.” The URLs of each data portal/tools are provided in the URL column.

The TCGA multi-omics datasets can be surveyed and studied by using different analysis and visualization tools/data portals (Figure 3A). A short description including the unique features, strengths, and limitations of the 10 most popular data portals and tools are given below:

### Genomic Data Commons (GDC)

**FIGURE 4:**
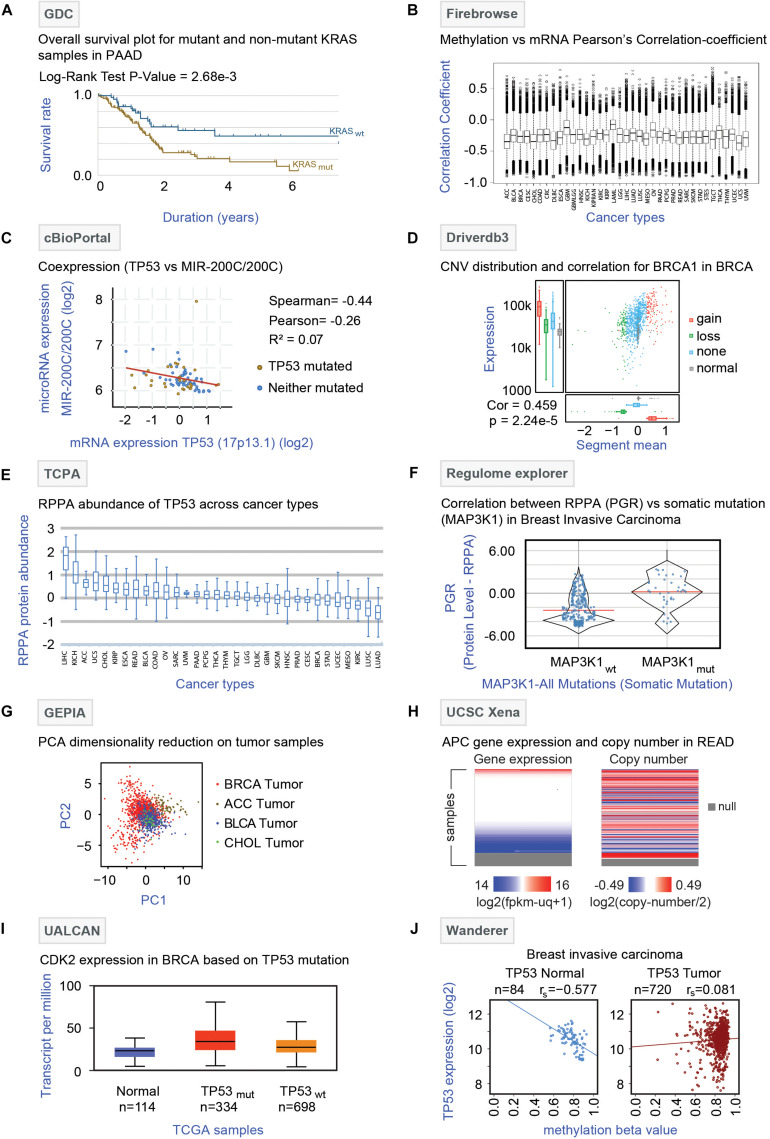
Analysis of TCGA multi-omics data by online tools. **(A)** GDC analysis: Survival analysis (Kaplan Meier curve) of pancreatic ductal adenocarcinoma (PAAD) patients with (KRAS_*mut*_, brown curve) or without (KRAS_*wt*_, blue curve) *KRAS* gene mutations is shown with log-rank *P*-value (2.68 × 10^–3^). **(B)** Firebrowse analysis: The differential correlation pattern between methylation (beta values) and mRNA expression (TPM) was obtained from Firebrowse and shown as a box plot across 37 different TCGA cancer types. **(C)** cBioPortal: Correlation plot depicting the correlation between the *TP53* gene and microRNA-200C expression is shown. The red line represents the regression slope. Spearman, Pearson, and Kendall rank correlation coefficients are given. **(D)** Driverdb3: Distribution of copy number variation (CNV) of the *BRCA1* gene in breast cancer (BRCA) samples is shown. The colors represent different forms of the *BRCA1* gene with or without CNV where red, green, blue, and gray indicate gain, loss, no-CNV, and normal, respectively. The box plots (left side) indicate the mRNA expression pattern of the different forms of *BRCA1*. The correlation between CNV and mRNA expression is given for the *BRCA1* gene. **(E)** TCPA: Differential abundance profiles of TP53 protein across 32 TCGA cancer types are shown as box plots. The *Y*-axis indicates the protein abundance as measured by the RPPA technique. The error bars represent the variation among the samples for a particular cancer type. **(F)** Regulome Explorer: The comparison of the protein abundance of progesterone receptor (PGR) between breast cancer (BRCA) patients with (MAP3K1_*mut*_) or without (MAP3K1_*wt*_) *MAP3K1* mutation is shown. The *Y*-axis represents protein abundance as identified by the RPPA technique. **(G)** GEPIA analysis: Dimensionality reduction through principal component analysis is shown. Four different tumor samples – BRCA, breast carcinoma; ACC, adrenocortical carcinoma; BLCA, bladder carcinoma; and CHOL, cholangiocarcinomas were plotted in two-dimensional planes of component 1 (PC1) and 2 (PC2) based on the mRNA expressions of 20 genes (*STARD3*, *CASC3*, *CLNS1A*, *MED1*, *LSM1*, *RPL19*, *BRF2*, *RPS6KB1*, *FADD*, *PSMD3*, *UBE2Z*, *PPFIA1*, *GRB7*, *RSF1*, *HEATR6*, *SNF8*, *ERBB2*, *ASH2L*, *WHSC1L1*, and *PHB*) for which the highest correlation between mRNA expression and methylation was found for breast cancer. The color represents each of the cancer type (Red: BRCA, brown: ACC, Blue: BLCA, and green: CHOL). **(H)** UCSC Xena: Gene expression and copy number alteration of *APC* gene in tumor samples of rectum adenocarcinoma (READ) patients are shown. The null section indicates a lack of data for those samples. **(I)** UALCAN: mRNA expression of the *CDK2* gene were plotted for normal and the breast cancer (TP53 mutant and TP53 non-mutant) samples. **(J)** Wanderer: *TP53* expression and methylation beta values were plotted for normal (*N* = 84) and breast cancer (*N* = 720) tissues. The correlation coefficient (r_*s*_) for each plot is indicated.

Genomic Data Commons (Jensen et al., 2017)^1^ is a repository of data mainly from TCGA but also includes data from the Tumor Alterations Relevant for Genomics-driven Therapy (TARGET) and Genomics Evidence Neoplasia Information Exchange (GENIE). GDC harbors raw multi-omics data, bio-specimen, and clinical resources of cancer patients. It provides dynamic data visualization options and analysis platforms enabling the creation of an adaptable interface that allows the users to filter data based on disease site, cancer-type, demographic data material, treatment, mutational impact, etc. The effects of somatic mutation patterns among highly mutated genes and comparison of demographic metadata can also be performed in this online portal. Additionally, GDC offers survival analysis by comparing the Kaplan-Meier survival plots between wild type and mutated cases of different genes. For instance, the survival curve of pancreatic ductal adenocarcinoma (PAAD) patients pre-stratified based on *KRAS* gene mutation is shown in Figure 4A. The analysis showed that patients with *KRAS* mutations have a poor prognosis compared to patients with wild-type *KRAS*. One major limitation is that a pan-cancer omics-comparison is not possible with the GDC data portal.

### Firebrowse

Firebrowse^2^ is a user-friendly interface for analyzing the reports originated through the Broad Institute’s TCGA-GDAC Firehose pipeline that contains the processed TCGA data. It serves as an excellent resource to download thousands of archive files and reports of TCGA data. In addition, it utilizes some graphical tools like viewGene (Kashuk et al., 2002) and iCoMut (Huang et al., 2019), to effectively explore mutation and gene expression patterns across various cancers in a genome-wide manner. Notably, for different TCGA cohorts, Firebrowse offers different analysis features for multi-omics data (mutation, mRNA-seq, miRNAseq, methylation, copy number, RPPA). Firebrowse can provide a list of genes that are associated with tumor stage, patient survival, gender, age, or ethnic background with respect to copy number alterations, methylation status, mRNA expression, and mutations (Zhang et al., 2018). The differential correlation pattern between methylation and mRNA expression obtained from Firebrowse across 37 different TCGA cancer types is shown in Figure 4B. Firebrowse does not host any datasets other than TCGA thereby restricting the integration of TCGA datasets to other data repositories.

### cBioPortal

cBioPortal (Cerami et al., 2012; Gao et al., 2013)^3^ is an open-access online resource developed at Memorial Sloan Kettering Cancer Center (MSKCC) for exploration and visualization of multi-omics data. The flexible interface of cBioPortal allows analysis of multiple data sets simultaneously and provides visualization features like-correlation plots for copy number alterations, mRNA expression, gene methylation, survival analysis (Kaplan–Meier plots), co-expression analysis, and network analysis (Klonowska et al., 2016). Notably, cBioPortal has two unique web tools- MutationMapper and OncoPrinter. MutationMapper is linked to 3D protein structure databases so that any user can understand the potential effects of the mutations with respect to proteins. On the other hand, OncoPrinter provides intuitive diagrams of genomic alterations such as somatic mutations and copy number alterations across a set of samples. As a representative analysis, the correlation plot highlighting the negative correlation between the *TP53* gene and microRNA-200C expression in glioblastoma generated by co-expression analysis is shown in Figure 4C. The major limitation of cBioPortal comes from the lack of omics-datasets from tumor-adjacent non-tumor (NT) tissues thereby restricting the tumor vs. non-tumor comparison.

### Driverdb3

Driverdb3 (Liu et al., 2020)^4^ is an online platform that utilizes diverse published algorithms for the identification of driver genes or mutations (Liu et al., 2020). This web portal has an innovative way of representing multi-omics events like- mutation profiling, expression levels, copy number variations (CNV), methylation status, and miRNA-gene network across different cancers types. Also for the cancer-specific driver genes, three levels of functional analysis (Gene Ontology, Pathways, and Protein/Genetics interactions) can be performed on this portal. Driverdb3 takes advantage of seven different public databases like KEGG, Reactome, PID, Biocarta, MsigDB, miRTar, and miRWalk for the pathway analysis. Furthermore, survival analysis (Kaplan-Meier plot) can be performed based on the mutations of a single or multiple genes in user-selected samples. An example of the major Driverdb3 analyses is shown in Figure 4D where CNV distribution and correlation to mRNA expression of the *BRCA1* gene are shown in breast cancer (BRCA) samples. The figure infers a positive correlation between CNV and expression levels through a scatterplot. Additionally, the highest mRNA expression level is found for tumor samples with copy number gain, whereas the lowest expression levels are observed for the normal samples. The major drawback of Driverdb3 is that it does not offer features to integrate TCGA derived molecular analysis with clinical features including tumor stages and location.

### The Cancer Proteome Atlas (TCPA)

The Cancer Proteome Atlas (Li et al., 2013, 2017)^5^ is a freely accessible data repository that focuses on the Reverse Phase Protein Array (RPPA) of TCGA tumor samples and cell lines. In order to make RPPA data accessible and analysis in a user-friendly manner, the TCPA web interface extensively offers a detailed analysis and visualization modules for RPPA data. By accessing RPPA data, the user can perform correlation analysis between proteins and find an association between protein abundance and prognosis in a patient-specific manner. Also, TCPA offers a comparison between two different types of tumors and enables the identification of proteins that are the most differentially expressed between tumor types. Additionally, RPPA-linked survival analysis, protein-drug analysis, and network-visualization modules are also provided by this portal. The differential abundance of TP53 protein in a pan-cancer manner as obtained by the TCPA database is shown in Figure 4E. The analysis showed that the highest expression of TP53 protein was observed in liver cancer (LIHC) followed by renal cancer (KICH) whereas the lowest protein expression was identified in lung cancer (lung adenocarcinoma – LUAD and lung squamous cell carcinoma – LUSC). TCPA does not harbor protein expression data from patient-matched non-tumor tissues; consequently, protein expression comparison between tumor vs. non-tumor tissues is not possible.

### Regulome Explorer

Regulome Explorer^6^ (Tomczak et al., 2015) is an integrative web platform where the interrelation between clinical and molecular features of TCGA samples can be explored. The unique feature of Regulome Explorer is the mapping of different data types to a circos plot with genomic coordinates. Using different tables and graphs, the associations between data types can be evaluated. Here users can filter data according to their parameters and visualize them. The algorithms used by this portal can perform correlation analysis between different genes (according to clinical/somatic copy number/gene expression/somatic mutation/methylation/miRNA expression/RPPA/tumor sample data) that can also be linked to pathway analysis. The comparison of the protein progesterone receptor (PGR) abundance between *MAP3K1* mutated and non-mutated samples in breast cancer (BRCA) is shown as an example (Figure 4F). This type of analysis can shed light on the indirect effect of the somatic mutations on gene and protein expression levels. Regulome Explorer lacks the patient-matched non-tumor omics-datasets.

### Gene Expression Profiling Interactive Analysis (GEPIA)

This online tool^7^ can be used for the rapid retrieval of customizable functionalities that depends on the data from The Genotype-Tissue Expression (GTEx) (Aguet et al., 2017) and TCGA. This website narrows down the gap between the bulk genomic data and their integrated processed information to the users in an intelligible way. This analysis platform allows the comparison of the expression profile of a gene of normal tissues (GTEx) to the corresponding tumors samples (TCGA) through dot plots or body maps. Differentially expressed genes and their chromosomal distributions can also be obtained by using different statistical approaches through this web tool. Some key functions that are included in this tool are profile plotting, correlation analysis, patient survival analysis, co-expression analysis, and dimensionality reduction analysis (Tang et al., 2017). As an example of dimensionality reduction through principal component analysis, Figure 4G shows different tumor samples (breast carcinoma: BRCA, adrenocortical carcinoma: ACC, bladder carcinoma: BLCA and cholangiocarcinomas: CHOL plotted in two-dimensional planes of component 1 (PC1) and 2 (PC2) based on the mRNA expressions of the genes for which the highest correlation between mRNA expression and methylation was found. One major limitation of GEPIA is that it does not allow to retrieve processed and normalized omics-data for TCGA samples.

### UCSC Xena

UCSC Xena^8^ (Goldman et al., 2018) allows users to explore public data resources such as - TCGA, GDC, ICGC, GTEx, TARGET, and PARADIGM pathway inference, along with individual studies involving somatic mutation, copy number, methylation, gene/protein expression, and phenotypic data. One of the key features of this tool is the comparability among the datasets facilitated by the visualization methods to identify the emerging patterns from multiple datasets. UCSC Xena provides the visualization of the gene expression, DNA copy number, methylation, and somatic mutational data for a user-defined gene and allows comparison across omics datasets, and thus offers the opportunity to establish a potential link between molecular signatures and clinical information across different cancer types. UCSC Xena additionally offers Kaplan-Meier analysis based on the genomic data for any subpopulation which can be visualized as high-resolution spreadsheets, scatter plots, bar charts, and box plots combined with statistical tests. For instance, gene expression and copy number alterations of the *APC* gene have been shown as a heatmap pattern for tumor samples of rectal cancer (READ) (Figure 4H).

### UALCAN

UALCAN (Chandrashekar et al., 2017)^9^ is a user-friendly web portal for analysis and visualization of the association between altered gene expression pattern, Kaplan-Meier based survival curves, of a particular TCGA cancer type. Users can compare the relative expression pattern of any given gene between tumor and non-tumor adjacent tissues in a paired analysis. These analyses can further be filtered based on different categories such as tumor grade, cancer stage, race, and other clinical features and the results can be exported in different output formats. Besides, up- or down-regulated genes for various cancer types can be identified through this portal. In summary, UALCAN has the potential to aid cancer researchers to identify the potential candidate biomarkers genes for diagnosis. As an example of UALCAN analysis, *CDK2* expression was plotted for normal, breast cancer (*TP53* mutant and *TP53* non-mutant) samples (Figure 4I). UALCAN web portal lacks the features that allow integration of TCGA datasets with other databases.

### Wanderer

Wanderer (Díez-Villanueva et al., 2015)^10^, an open-access web server, offers exploration and interpretation of gene-specific expression profiles and DNA methylation patterns for almost all of the cancer types available in TCGA. Users can inquire about a gene of interest on a specific cohort of TCGA and the gene expression in specified exon regions along with their gene locations and CpG islands can also be investigated through different graphical outputs. Furthermore, it offers the exploration of the DNA methylation alterations in a gene-specific manner. Besides, normal–tumor paired comparisons through comprehensive tables and graphs as well as correlation analysis can be performed. As an example, *TP53* expression and methylation beta values were plotted for normal and breast cancer tissue (Figure 4J). Wanderer lacks pan-cancer and clinical data analysis features.

The comparison of these TCGA-associated web portals and tools with respect to their relative strengths and limitations is shown in Figure 3B.

### International Cancer Genome Consortium (ICGC)

The International Cancer Genome Consortium (ICGC) is a comprehensive repository for cancer-specific multi-omics datasets encompassing 90 different cancer projects involving 16 different countries. Out of these 90 projects, omics-datasets for 86 projects are available through the ICGC portal spanning 35 tumor types including 22 primary cancer sites. Among the 86 projects, 24 projects led by the TCGA consortium are based in the United States, focusing mainly on Caucasian, non-Hispanic white-American, followed by African-American, and Hispanic or Latino ethnic groups. The rest of the projects (*N* = 62) are based on samples obtained from 15 different countries around the world. ICGC provides genomic, transcriptomic, and epigenomic datasets of specific cancer types and subtypes thereby aiding the analysis of simple and structural somatic mutations, germline variations, array/sequence-based DNA methylation pattern, structural rearrangements of chromosomes, and gene/miRNA expression. Since its establishment in 2007, ICGC holds a massive collection of omics-data (1.69 petabytes) from a total of 24,289 donors across the world that is kept up to date.

**FIGURE 5:**
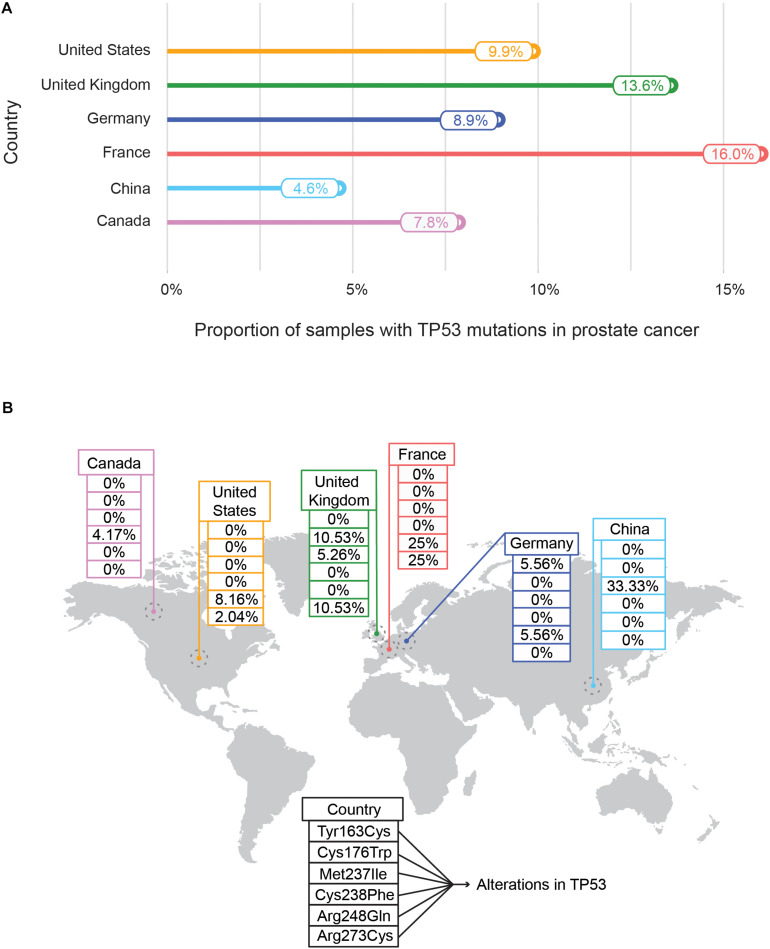
Mutational frequencies **(A)** and distribution of mutational hotspots **(B)** of TP53 in prostate cancer across different countries. **(A)** Represents the mutational frequencies of *TP53* in prostate cancer as obtained from the International Cancer Genome Consortium (ICGC) data portal were plotted across different countries – United States, United Kingdom, Germany, France, China, and Canada. The mutational frequency is defined as the proportion of prostate tissue samples with *TP53* mutations with respect to total prostate tissue samples collected. **(B)** Illustrates the distribution of *TP53* mutational hotspots along with their frequencies in prostate cancer samples across countries. In total six mutational hotspots are indicated (Tyr163Cys, Met237Ile, Arg273Cys, Arg248Gln, Cys238Phe, and Cys176Trp).

Recently an international collaboration based pan-cancer project of ICGC and TCGA called Pan-Cancer Analysis of Whole Genomes Consortium (PCAWG) examined the features and consequences of genomic variations by focusing on both coding and non-coding regions of 2,658 whole-cancer genomes (Campbell et al., 2020). ICGC also highlights the genome-wide mutational landscape analysis of genes for a specific cancer type across different countries. As an example, to highlight the population-wise mutational frequency of genes, we selected the somatic mutational events in *TP53* – a tumor suppressor gene. The comparison of different mutational frequencies of *TP53* in prostate cancer among six different countries is shown in Figure 5A. The highest mutation rate of the *TP53* gene has been assigned to France (16.0%) followed by the United Kingdom (13.6%) whereas the lowest has been observed in Canada (7.8%) and China (4.9%) indicating the population-wise differences of the mutational burden of TP53 (Figure 5A). The other countries (United States: 9.9%, and Germany: 8.9%) showed an intermediate range of mutational events in *TP53*. Moreover, the distributions of six different hotspot variants (Tyr163Cys, Cys176Trp, Met237Ile, Cys238Phe, Arg248Gln, and Arg273Cys) of *TP53* with the highest frequency in prostate cancer for different countries are shown (Figure 5B). Two mutational hotspots (Arg248Gln and Arg273Cys) with equal frequency (25%) were observed in France. In contrast, a different mutational hotspot (Met237Ile) with a frequency of 33.33% was identified in China (Figure 5B). This variation of mutational hotspots underscores the role of genomic diversity across divergent populations in determining the population-specific propensities of mutational hotspots of a particular gene.

In ICGC, the highest number of samples (apart from TCGA samples) with available molecular data is deposited for neuroblastoma (NBL-US, *N* = 798), followed by acute lymphoblastic leukemia (ALL-US, *N* = 615). In contrast, the lowest number of samples is for liver cancer (LIHM-FR, *N* = 4), renal cancer (RECA-CN, *N* = 10), and Lung cancer (LUSC-CN, *N* = 10). Although ICGC holds a huge amount of cancer-specific genomic, epigenomic and transcriptomic datasets, not all omics-data types are available for different populations of the world. For instance, apart from the thirteen projects in collaboration with TCGA, no other projects have protein expression data. Similarly, besides TCGA projects, only two projects (Malignant lymphoma- Germany and Ovarian cancer- Australia) have miRNA expression data. This lack of pervasiveness of multi-omics data can potentially pose an impediment to the ongoing efforts to understand cancer pathogenesis in a population-wise manner.

## Cancer-Specific Single-Omics Resources

This category represents the resources that harbor cancer-specific single-omics datasets such as genomics and proteomics data.

### Catalogue of Somatic Mutations in Cancer (COSMIC)

COSMIC^11^ represents one of the largest sources of manually curated catalogs of somatic mutation across different types of human cancers (Tate et al., 2019). The source of the COSMIC database can broadly be divided into two main types – manually curated high precision datasets and genome-wide screening datasets. High precision datasets are obtained from manual interpretation from over 26,000 peer-reviewed publications by COSMIC’s team focusing on well-characterized driver genes known as The Cancer Gene Census (CGC) for which the mechanistic links to cancer have been established (Sondka et al., 2018). CGC is divided into two groups (tiers) based on their documentation. For instance, Tier-1 genes must have a well-documented and established relevance to cancer as well as corresponding mutational evidence in the cancerous transformation that is supported by a broad literature base. Tier-1 CGC includes mutations in the tumor suppressor genes (TSGs) and oncogenes where the former typically are subjected to inactivating mutations and the latter serve as hotspots for missense mutations (Sondka et al., 2018). In addition, genes with oncogenic fusions are also included in Tier-1 if their altered functions drive oncogenesis or serve as regulatory elements for other proteins. Whereas, in Tier 2, genes that recently have emerged to have a strong manifestation in cancer without extensive available evidence and confirmed roles are included (Sondka et al., 2018). CGC provides simple graphics to depict the function of cancer-associated genes in the attainment of cancer-hallmarks (Sondka et al., 2018). COSMIC aims to identify genes associated with cancer-promoting or cancer-suppressing functions and relate them to cancer-hallmarks by presenting the summary of the relevant information and facts with access to the literature base. In addition to the CGC, COSMIC also harbors datasets originated from 32,000 genome-wide screening studies and datasets from other repositories such as TCGA and ICGC. This genome-wide screening represents unbiased global mutational landscapes of cancer-genomes (Tate et al., 2019).

**FIGURE 6:**
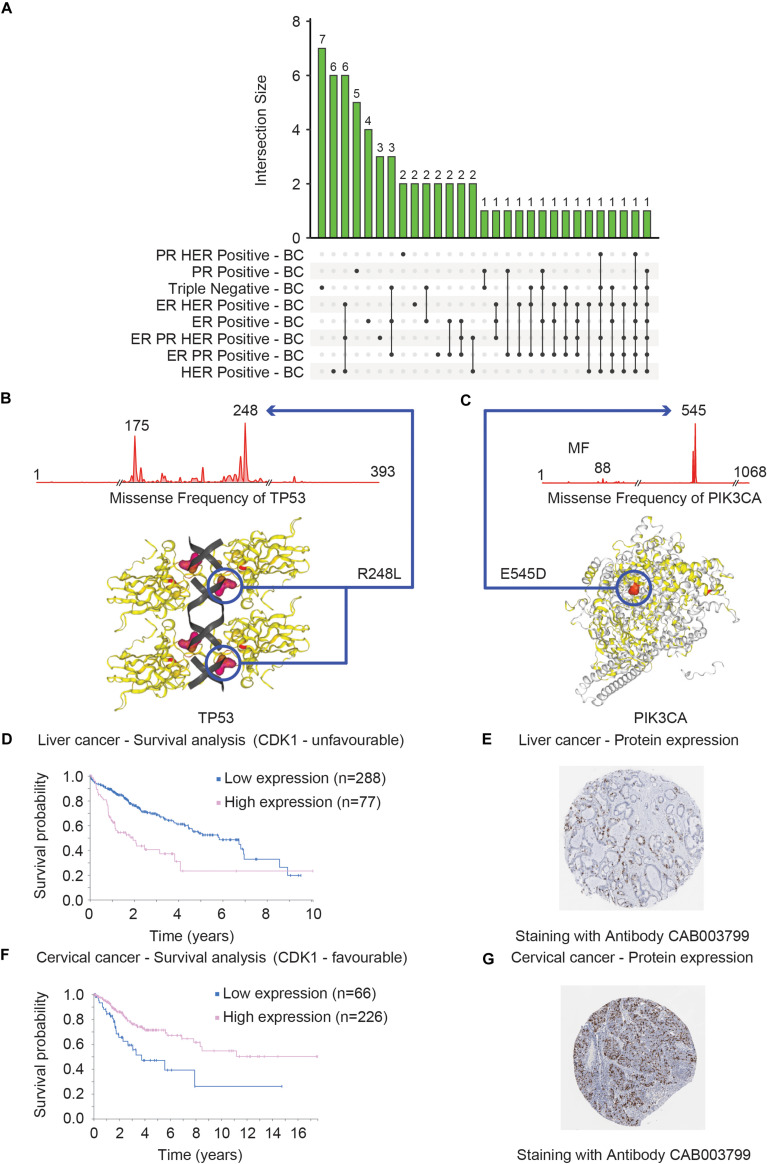
Clinical and structural consequences of mutational events of *TP53*, *PIK3CA*, and *CDK1* genes. **(A)** Shows that distribution of the top 20 mutated genes across eight different breast cancer subtypes (ER PR Positive – BC, ER PR HER Positive – BC, ER Positive – BC, ER HER Positive – BC, Triple Negative – BC, PR Positive – BC, PR HER Positive – BC and HER Positive – BC) in the form of Upset Venn diagram. The Venn diagram was based on the data obtained from COSMIC. Two genes – *TP53* and *PIK3CA* were found to be commonly mutated in seven of the eight subtypes (represented by the last two vertical bars). The most commonly mutated residues in *TP53* and *PIK3CA* are R248L **(B)** and E545D **(C)**, respectively, in triple-negative breast cancer. The consequences of these mutations on the 3D structure that can consequently alter the function of TP53 **(B)** and PIK3CA **(C)** proteins are shown. The survival curves of the liver **(D)** and cervical **(F)** cancer patients with high and low expression of *CDK1* are shown. These plots were generated by The Pathology Atlas. High *CDK1* expression was annotated as unfavorable **(D)** and favorable in terms of prognosis for liver and cervical **(F)** cancer. The protein levels validation of CDK1 expression in liver **(E)** and cervical **(G)** cancer tissues measured by the IHC technique are shown. The antibodies used for the IHC stating are indicated for both cancer tissues.

Overall COSMIC offers the exploration of genomic data focusing on mutational types and frequency statistics for a user-defined gene or cancer type. For instance, by choosing different subtypes of breast cancer (BC), the output shows the top-20 most frequently mutated genes in a particular BC subtype. Figure 6A shows the common and exclusive genes that are commonly mutated in different BC subtypes.

In addition, COSMIC provides a graphical representation of the mutational frequencies of a given gene in a particular cancer type. For instance, the missense mutations of *TP53* (R248L) (Figure 6B) and *PIK3CA* (E545D) (Figure 6C) have the highest occurrence frequency in triple-negative BC. The COSMIC-3D feature adds an additional but very important layer of information to mutational data by allowing the user to map these mutations onto the 3D structure of the selected protein (Jubb et al., 2018). These features aid users to evaluate the impact of missense mutations on the protein structure as well as the interaction between protein and small molecules and facilitate the prediction of functional consequences of the mutational events (Figures 6B,C). Overall, the COSMIC database and corresponding analysis tools provide an extensive exploration opportunity of the somatic mutational landscape of the cancer genome. One of the major limitations is that COSMIC does not offer the features to integrate mutational datasets to other data types such as mRNA expression profiles.

### The Pathology Atlas

The pathology atlas is a part of the Human Protein Atlas (HPA)^12^ – a platform of data repository including antibody-based imaging, mass spectrometry-based proteomics, and transcriptomics (Uhlén et al., 2015). HPA allows the data integration approach to map the proteins in human cells, tissues, and organs for human proteome exploration in greater detail. Among the different segments of HPA, The Pathology Atlas is dedicated to analyzing the altered mRNA and protein levels in cancer states including a correlation pattern of mRNA and protein levels with the survival of cancer patients (Uhlen et al., 2017). The Pathology Atlas consists of the detailed analyses of protein-immunohistochemistry, mass-spectrometry, and TCGA-derived transcriptome data of 17 different cancer types from 8000 patients. To harness the translational potential of the omics analysis, The Pathology Atlas provides more than 400,000 interactive survival scatter plots delineating the consequence of altered mRNA and protein levels on the survival of cancer patients. While the transcriptomics data has been retrieved from the Cancer Genome Atlas, the unique feature of The Pathology Atlas includes five million pathology-based images obtained through tissue microarray (TMA)-based immunohistochemistry (IHC) analysis of the corresponding proteins. In search of the important clues associated with cancer, the HPA consortium analyzed the transcriptome in 17 different cancer types of TCGA and correlated the altered-gene expression to clinical outcomes. Uniquely, they attempted to validate the genes with prognostic potential at the protein level, by performing IHC analyses of tumor tissues (*n* = 357). The IHC analysis validated the prognostic potential of these genes at the protein level including the endoplasmic reticulum oxidoreductase α protein ERO1A and two proteins – S100A10 and S100A16 belonging to the S100 family. S100A10 and S100A16 proteins were confirmed to have strong prognostic potential in the NSCLC cohort including adenocarcinomas and squamous cell carcinomas (Uhlen et al., 2017). The example of a proliferation marker – MKI67 highlights the utility of The Pathology Atlas for validating the prognostic potential of candidate markers. The clinical application of MKI67 has been suggested but its prognostic potential was controversial (Penault-Llorca and Radosevic-Robin, 2017). However, in The Pathology Atlas study, MKI67 was found not to be associated with prognosis in the NSCLC cohort (Uhlen et al., 2017). As a representative analysis, a survival plot was generated for the liver (Figure 6D) and cervical (Figure 6F) cancer patients with a high and low *CDK1* expression, respectively. The survival analysis showed that high *CDK1* expression has lower survival probability in liver cancer patients compared to patients with low *CDK1* expression and thus *CDK1* was classified as unfavorable while, in cervical cancer the high expression of the same gene *CDK1* was associated with relatively higher survival probability compared to low expression of *CDK1*. The protein level evidence of CDK1 in the liver (Figure 6E) and cervical (Figure 6G) cancer as generated by the IHC method are shown. In summary, The Pathology Atlas confirmed the protein-level prognostic values of targeted genes in lung, renal, pancreatic, and liver cancers, and the analysis of the independent lung cohort (Uhlen et al., 2017). One of the downsides of The Pathology Atlas is that it does not offer integrated analysis platforms of mRNA and protein-level data.

## Generalized Single-Omics Data Resources

In this section, we classified the single-omics – transcriptomics and proteomics data repositories that harbor omics-data for various organisms and a multitude of disease-associated conditions including cancer-specific datasets.

### Gene Expression Omnibus (GEO)

**FIGURE 7:**
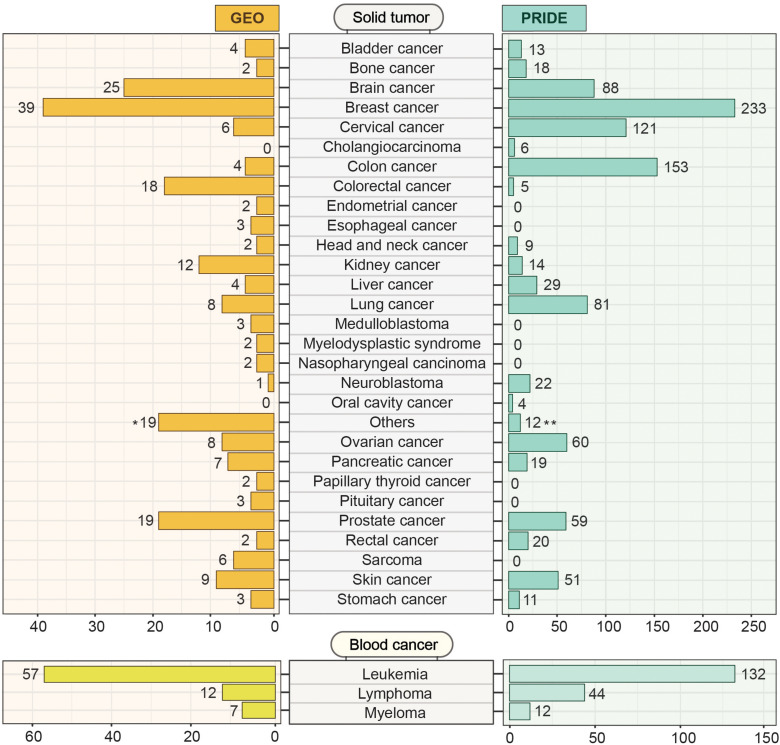
Cancer-specific transcriptomics and proteomics datasets deposited in GEO and PRIDE. GEO and PRIDE repositories were systematically mined for cancer-specific transcriptomics and proteomics datasets. The mined datasets were then classified into solid tumor and blood cancer categories. Datasets from different studies within each category representing a particular solid tumor or blood cancer type were indicated. The number in brackets indicates the number of studies for which the datasets were deposited. The Others (19)* category on the GEO section represents 19 different types of cancer (Adenoid cystic carcinoma, Adrenocortical adenomas, Aldosterone-producing adenoma, Alveolar rhabdomyosarcoma, Anaplastic thyroid carcinomas, Gastrointestinal cancer, Hypothalamic hamartomas, Liposarcoma, Malignant pleural mesothelioma, Neuroectodermal tumors, Oral squamous cell carcinoma, Pediatric malignant germ cell tumors, Pheochromocytomas, Prolactinomas, Retinoblastomas, Testicular seminoma, Uterine smooth muscle tumor, Uveal melanoma, Waldenstrom’s macroglobulinemia) holding one dataset each. The Others (12)** category on the PRIDE section contains 12 general studies related to cancer.

Gene Expression Omnibus^13^ is an open-access data resource driven by user-uploaded datasets. As of May 2020, GEO harbors 4348 datasets derived from more than 100 different organisms. This huge volume of data can effectively be accessed and explored using user-friendly web-based tools. While GEO hosts all sorts of data including cell line and non-cancer samples, we systematically mined the portal using MeSH (Medical Subject Headings) terms to identify 291 patient datasets encompassing 48 different cancer types (Figure 7). Among the solid tumor types, breast cancer, and brain cancer (including Glioma, Medulloblastoma, Glioblastoma, and Glial brain tumor) are the prevalent categories having 39 and 25 independent projects, respectively. In the case of blood cancers, GEO offers 57 independent studies on leukemia, 12 on lymphoma, and 7 on myeloma. With all the transcriptome datasets, GEO has emerged as a comprehensive platform for large, annotated compendia of gene expression profiles across tumor tissues and provides the opportunity to integrate these transcriptomics datasets with other omics levels. In GEO, the unavailability of the features to integrate different expression datasets from multiple studies restricts the cross-dataset analysis.

### Proteomics IDEntifications Database (PRIDE)

Originated from the European Bioinformatics Institute (EBI, Cambridge, United Kingdom), the PRoteomics IDEntifications (PRIDE) database^14^ hosts a massive amount of high-throughput mass-spectrometry (LC-MS/MS) based proteomics resources and datasets. By May 2020, PRIDE has accumulated around 457 million peptide-spectra for several organisms from 64 different countries across the world. These datasets include disease-free samples as well as samples from disease conditions like- Parkinson’s disease, cardiovascular disorder, diabetes mellitus, and cancer. Cancer-specific LC-MS/MS datasets along with result outputs that are deposited in the PRIDE database can be explored and retrieved for further analysis. Figure 7 represents the number of datasets from PRIDE representing different cancer types. We systematically searched for cancer datasets by g disease filter into the search bar and the results output is then analyzed after retrieval. Among the 1028 proteomics studies focusing on different types of solid tumors, breast cancer has the highest number (*N* = 233) of datasets (Figure 7). In contrast, with 188 proteomics studies, leukemia has the highest number of studies among different types of blood cancer studies. The major limitations of PRIDE include the unavailability of clinical data. Although PRIDE offers some data analysis tools like PRIDE inspector which is useful to inspect the raw spectra, it lacks the features to analyze (e.g., Differential expression analysis) and visualize protein-level data.

## Strategies for Multi-Omics Data Integration

**FIGURE 8:**
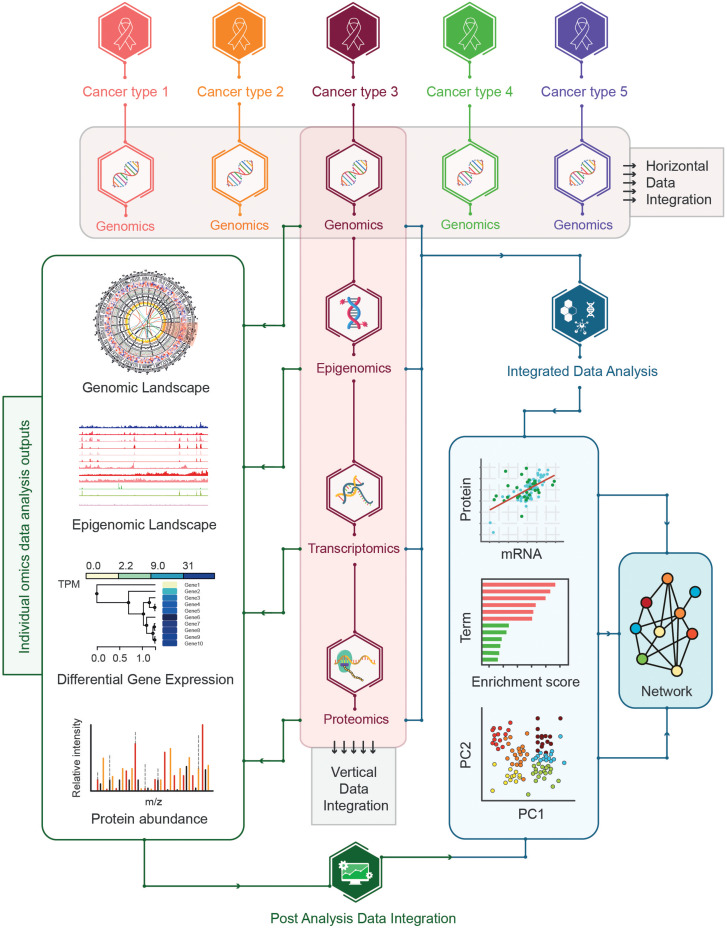
Strategies for multi-omics data integration. The horizontal and vertical data integration strategies are shown where horizontal integration represents the integration of single omics across multiple cancer types whereas vertical integration encompasses the integration of multi-omics data for the same cancer types. Additional vertical integration can be further divided into post-analysis integration and integrated data-analysis strategies. In post analysis integration individual omics data is analyzed in isolation before combining the pre-analyzed multi-omics datasets whereas the integrated analysis represents the integration of multi-omics datasets followed by the analysis of integrated datasets.

The integration of multi-omics data for cancer samples encompassing the co-analysis of multi-omics data including genomics, epigenomics, transcriptomics, and proteomics can be challenging. Given the inherent dissimilarities of different types of omics data, a wide range of skills and expertise is required to investigate the feasibility of the integration of multi-omics data across different platforms. A number of approaches have been proposed for integrating omics-data such as horizontal data integration involving the integration of a single level omics-data across different studies and vertical data integration representing the integration of different types of omics-data for the same type of samples (Wu et al., 2019; Figure 8).

## Horizontal Data Integration

A prominent example of the horizontal data integration of 2,658 whole-cancer genomes and their matching normal tissues across 38 tumor types was performed by the Pan-Cancer Analysis of Whole Genomes (PCAWG) Consortium (Campbell et al., 2020). PCAWG uncovered that the majority of the tumors (91%) harbored at least one well-characterized driver mutation, with an average of 4.6 driver mutations per tumor. Furthermore, PCAWG identified that chromothripsis – an event where clustered structural variants originate at a single time point, occurs in the early phase of tumor evolution in melanoma, and subsequently affect cancer-associated genes. However, the lack of other omics-analysis restricted the exploration of these structural variants on the transcriptomic and proteomic landscapes (Campbell et al., 2020). Horizontal data integration strategies such as PCAWG are well suited to investigate the origins of cancerous transformation and the evolution of tumors in terms of clonal and subclonal genomic alterations (Campbell et al., 2020). However, by design, horizontal data integration is focused on a particular omics type (genomics in case of PCAWG) that makes them limited in exploring the effect of genomic aberration events on other omics levels and may not be a suitable approach to stratifying patients and discovering biomarkers.

## Vertical Data Integration

Proteogenomics analyses (integration of genomics, epigenomics, transcriptomics, and proteomics) of particular cancer types (Zhang et al., 2014, 2016; Mertins et al., 2016) can be an example of vertical data integration. The advantages of this method include exploration of the multidimensionality of tumor cells in terms of molecular association of multi-omics levels that may lead to the identification of new molecular features, genotype to phenotype correlation, patient-stratification, and biomarker discovery. For instance, one of the applications of integrating proteomics and genomics (proteogenomic approach) includes the identification of the polypeptides encoded by long non-coding RNAs (LncRNAs) in colon and prostate tumor tissues (Chakraborty et al., 2019). In colorectal cancer, by integrating mutation, copy number, methylation, mRNA, microRNA, and proteomics datasets, Guinney et al. uncovered four consensus molecular subtypes (CMSs) that are more aligned with the clinical stratification and thus may facilitate the CMS-subtype based targeted interventions (Guinney et al., 2015). Furthermore, for breast cancer, the proteogenomic approach has proven to be beneficial in the identification of possible druggable targets- CDK12, TLK2, PAK1, and RIPK2 (Mertins et al., 2016). For high-grade serous ovarian cancer (HGSC), the abundance profiles of proteins belonging to cell invasion and migration were found to be modulated by copy number alterations (CNA) indicating a possible role of CNA-driven proteogenomic events in attaining these hallmarks of cancer (Zhang et al., 2016). However, the application of the proteogenomic approach to multiple cancer types is challenging. The complexity of generating multi-omics datasets in a pan-cancer manner remains one of the hurdles to materialize the potential of proteogenomics application to multiple cancer types.

In addition, a different approach has been adopted to classify the vertical data integration strategies based on pre- and post-analyzed omics-data integration. In this approach, vertical data integration strategies can be further divided into two categories: post-analysis data integration, and integrated data analysis (Pinu et al., 2019; Figure 8).

## Post-Analysis Data Integration

Post-analysis data integration includes analysis of single omics datasets individually followed by their integration by focusing on key overlapping features that are networked together to identify the alteration of biological pathways that are perturbed by a certain condition. For instance, in pediatric acute lymphoblastic leukemia, methylome and transcriptome datasets were analyzed separately to identify differentially methylated (DMGs) and differentially expressed genes (DEGs) followed by their integration revealed that the gene expression and methylation alterations occur in the same molecular pathways (Ras signaling pathway, PI3K-Akt signaling pathway, and Rap1 signaling pathway) (Sanchez and Mackenzie, 2020). However, post-analysis data integration fails to capture the latent multi-modal molecular signatures across different omics levels.

## Integrated Data Analysis

In contrast to post-analysis integration, the integrated data analysis approach takes advantage of specialized algorithms and tools to combine pre-analyzed data sets across different omics platforms before committing to data analysis. For instance, an integrated analysis of the proteomic and transcriptomic data representing 65 breast cancer (BC) and 53 adjacent non-cancerous tissues revealed a global higher mRNA-to-protein concordance in tumors where the increased concordance between mRNA and protein levels was linked with aggressive BC, including basal-like/triple-negative BC, and decreased survival (Tang et al., 2018). Despite the relative strengths and weaknesses of post-analysis and integrated approaches, these strategies are currently being used as exploratory tools to generate a new hypothesis or to provide some mechanistic explanation of cancer phenotypes.

## Algorithms of Data-Integration

The algorithms for data-integration can broadly be classified into five categories: network-based, Bayesian, fusion-based, similarity-based, correlation-based, and other multivariate methods (Subramanian et al., 2020). The comparison among the different algorithms with respect to their methods and applicability has been shown in Table 4.

**TABLE 4:** Comparison of different multi-omics integration algorithms with respect to methods and applications.

Algorithms	Methods	Applications		
		Cancer subtyping	Cancer biomarker prediction	Cancer mechanisms
SNF	Network-based	+	–	–
iOmicsPASS	Network-based	+	+	+
iClusterPlus	Bayesian	+	+	+
PFA	Fusion-based	+	–	–
NEMO	Similarity-based	+	–	–
CCA	Correlation-based	–	–	+
moCluster	Multivariate analysis	+	-	–

*SNF, Similarity network fusion; PFA, Pattern fusion analysis; NEMO, NEighborhood based multi-omics clustering; CCA, canonical correlation analysis; + indicates the appropriateness of the algorithm; – indicates the inappropriateness of the algorithm.*

### Similarity Network Fusion (SNF)

Similarity network fusion is a network-based approach for the integrative multi-omics data sets. SNF involves the construction of the individual network of patient samples from each available omics-data type followed by the fusion of the individual networks into a single network representing all omics-datasets. For instance, Wang et al. (2014) employed SNF on DNA methylation, mRNA expression, and miRNA expression across 215 patients with glioblastoma (GBM). Individual data types resulted in a different pattern than all three data types that were fused. DNA methylation and mRNA expression data analysis individually resulted in the smallest and medium-sized patient clusters, respectively. In contrast, the fused network yielded a much clearer pattern of clustering where the smallest cluster (subtype-3) is composed of younger patients with the IDH subtype with a more favorable prognosis and cluster 1 (subtype-1) patients exhibited a positive response to drug temozolomide (TMZ) (Wang et al., 2014). Although SNF is suitable for distinguishing different subtypes of a particular cancer type, it may not be applicable to identify biomarkers or provide mechanistic insight to cancer phenotypes.

## iOmicsPass

Another network-based proposition iOmicsPASS developed by Koh (2019) performs a supervised analysis of quantitative multi-omics data by computing biological interaction scores for the user given network. This model uses a shrunken gene-centroid algorithm to the resulting interaction scores to select the best predictive sub-networks for breast cancer (BC) phenotypic groups. Apart from identifying the distinct molecular signatures specifying the different phenotypic groups, iOmicsPASS analysis also uncovered the protein markers as well as the underlying transcriptional regulatory circuits for the basal-like BC subtype.

## iClusterPlus

To extend the breadth of omics-data integration, Mo et al. (2013) proposed a framework known as iClusterPlus involving a model selection process relying on a Bayesian information criterion to integrate multi-omics data. By utilizing the iClusterPlus algorithm, the authors modeled discrete and continuous variables originating from integrated genomic, epigenomic, and transcriptomic datasets from 189 TCGA colorectal carcinoma samples. The authors were able to identify an intermediate chromosomal instability phenotype in addition to the previously characterized chromosomally stable or unstable subtypes (Mo et al., 2013).

### Pattern Fusion Analysis (PFA)

The PFA framework established by Shi et al. can perform information-alignment and bias correction for the fusion local sample-patterns originating from each dataset into a global sample-pattern corresponding to phenotypes in an automated manner. Applying PFA on the gene expression, miRNA expression, and DNA methylation profiles of the TCGA samples from clear cell carcinoma (KIRC), lung squamous cell carcinoma (LUSC), and glioblastoma (GBM) resulted in clustering patterns that were similar to SNF and iCluster but with higher clinical prognosis efficiency (Shi et al., 2017). PFA may not be suitable for biomarker discovery and gaining mechanistic insights into cancer phenotypes.

### NEighborhood Based Multi-Omics Clustering (NEMO)

One of the major challenges in the integration of multi-omics data is the partial dataset representing the lack of completeness of omics-data for each sample across different omics platforms as is the case in TCGA. AML cohort (*N* = 197) from TCGA can be considered as an example of a partial dataset, encompassing 173 patients with mRNA expression profiles, 194 with methylation, and 188 with miRNA expression profiles. Due to their partial nature, the three omics datasets cannot be directly clustered using conventional algorithms rather the clustering must be restricted to a sub-cohort of 170 patients with all three omics-data levels or imputations can be performed to replace the missing values (Rappoport et al., 2019). To circumvent the challenges of partial omics-data, a similarity-based multi-omics clustering approach known as NEMO was developed by Rappoport and Shamir, Rappoport et al. (2019). NEMO appears to perform clustering analysis that is highly correlated with prognosis using partial multi-omics datasets from TCGA AML samples without imputation or reducing sample numbers (Rappoport et al., 2019). However, the identification of novel biomarkers may not be possible with NEMO.

### Canonical Correlation Analysis (CCA)

A correlation-based method such as the canonical correlation analysis (CCA) is typically used to explore the extent of correlation across copy number, methylation, and gene-expression (Lin et al., 2013; Zhou et al., 2015). Although CCA-derived co-expression networks can provide important molecular insight into biological processes and mechanisms of carcinogenesis (Hong et al., 2013), CCA has low applicability in distinguishing disease-subtypes and identifying biomarkers.

### moCluster

A multivariate analysis based platform known as moCluster that employs the multivariate analysis method that was used to identify the latent patterns across DNA methylation, gene expression, and protein expression data from 83 samples of colorectal cancer from TCGA and CPTAC (Meng et al., 2016). Integration of methylation, mRNA, and protein data from colorectal cancer patients using moCluster identified four molecular subtypes, including one with microsatellite instability and upregulation of immune-system related genes/proteins such as PDL1 (Meng et al., 2016). The other three subtypes were not previously discovered using single data sets, which strongly indicated that integrated approaches are needed to untangle the molecular complexity of carcinogenesis (Meng et al., 2016). Although moCluster was effective in identifying the latent molecular subtypes, it may not be suitable to uncover the underlying mechanisms behind the phenotypic characteristics of the molecular subtypes.

## Current Challenges of Multi-Omics Data Integration

One of the major challenges in cancer research comes from selecting the appropriate control to compare the cancer-specific aberrations in omics levels. Insights gained from omics-data integration to understand cancer biology relies on the comparison between healthy and cancer samples under the assumption that the difference may be directly related to cancer. However, comparison of omics-data between healthy individuals and cancer patients to identify distinct omics-signatures that are associated to complex phenotypes such as cancer hallmarks can be challenging due to the variability with respect to many confounding factors like inter-individual genomic diversity, the cell-type composition of the tissue, and other technical factors (Hasin et al., 2017). For instance, the modulatory role of inter-individual genomic diversity on gene expression has been revealed by a seminal study involving the deep-survey of gene expression across 44 human normal tissue types encompassing 7,051 different samples from 449 donors performed by The GTEx (Genotype-Tissue Expression) Consortium (Aguet et al., 2017). In search of the regulatory influence of the genetic variation on gene expression, the project identified that the expression of most genes are regulated by genetic variations that are located within 1 Mb of the target gene’s transcription start site (TSS) (Aguet et al., 2017). However, applying a statistical model may take confounding factors into account to identify more accurate cancer-specific molecular signatures. The challenges of inherent-genomic variability between cancer patients and healthy individuals can additionally be overcome, by considering histologically normal samples adjacent to the tumor commonly known as normal adjacent to tumor (NT), as a healthy control in the case of solid tumors as mentioned before. However, the use of NT as healthy control samples has been controversial as one of the recent studies involving 6506 samples across eight cancer, NT counterparts, and corresponding healthy tissue types showed that NT represents an intermediate between healthy and tumor tissues (Aran et al., 2017). Moreover, for most cancer types, not all omics data types are generated in the majority of the NT or blood-derived normal (NB) samples resulting in only a subset of samples with complete multi-omics datasets (Figure 2). Algorithms such as NEMO (described earlier) have been proven to facilitate the analysis of the partial multi-omics data sets (data for only a subset of the samples) without losing the statistical power (Rappoport et al., 2019). For a solid-tumor transcriptomics study, a lower number or the absence of the normal samples can additionally be compensated for by including data from GTEx samples, to be compared with cancer. GEPIA takes advantage of TCGA and GTEx mRNA-seq datasets to explore differential gene expression patterns between cancer and normal states that can be correlated to pathological stages, patient survival analysis, correlation analysis, and PCA-based dimensionality reduction analysis (Tang et al., 2017).

Tumor heterogeneity poses another significant challenge in the integrative multi-omics analysis. Most tumor tissues are composed of a diverse set of tumor cell subpopulations with distinct genomic and transcriptional signatures. This heterogeneity typically emerges from random genetic mutations that take place during the rapid proliferation of cancer cells. In-depth characterization of these subpopulations thus becomes an important factor to unlock the levels of tumor heterogeneity and essential to design a treatment regimen to ensure the success of anti-cancer treatments. The recent advent of single-cell sequencing technologies provides a new platform to characterize the tumor heterogeneity in greater detail. **Single-cell DNA-seq** technologies have higher sensitivity to detect the minority clones among tumor populations thus providing a genomic level characterization of tumor heterogeneity. On the other hand, **single-cell RNA-seq** that are used to map the transcriptional landscape of different subpopulations within a tumor can lead to a greater understanding of cancer progression mechanisms. However, single-cell technologies to study altered LC-MS/MS-based proteomics have been not possible until now and so far in a typical single mammalian cell, accurate analysis has only been possible for the most abundant proteins. An alternative antibody-based approach to profile targeted protein profiling is also being increasingly applied on a broader scale. The technique known as **mass cytometry** is based on the pre-incubation of cells with antibodies that are conjugated to stable heavy metal isotopes followed by the passage of cells into a nebulizer that gives rise to single-cell droplets into a mass cytometer (Spitzer and Nolan, 2016). However, as with any antibody-based method mass-cytometry is more restricted to available antibodies and only known proteins can be targeted therefore making it unsuitable for global exploratory analysis (Spitzer and Nolan, 2016). Attempts are being taken to develop robust and accurate techniques for single-cell proteomics methods with higher sensitivity (Marx, 2019).

## Future of Cancer-Research: Multi-Omics Driven Systems Biology Approach for Precision Medicine

The burgeoning of NGS and mass-spectrometric techniques equipped with powerful computational tools has made it possible to integrate multi-omics data to uncover the link among the altered molecular-signatures across different omics-levels. The interconnected molecular-signatures of multi-omics data provide an opportunity to understand cellular response on the systems level. The online omics-data resources provide a unique opportunity to integrate multi-omics data through a systems biology approach, but at the same time pose a huge challenge to model thousands of genes, mRNAs, and proteins alterations in an adaptive manner. The systems biology approach is based on the development of predictive models that are continuously refined and validated by experimental data. One of the major goals of the systems biology approach is to identify the key molecular features that are associated with cellular-phenotypic consequences (Adlung et al., 2017). These predictive models are assumed to be particularly beneficial to stratify patients based on distinct molecular signatures to determine who are most likely to benefit from targeted therapies (Guhathakurta et al., 2013). The translational applications of systems biology driven predictive-models have already been demonstrated for drug-resistance and targeted therapy. A prominent example is uncovering the resistance mechanism of trastuzumab – a recombinant humanized monoclonal antibody that binds with the human epidermal growth factor receptor protein (HER2) in HER2+ breast cancer (BC) patients. Systems biology driven models coupled with whole-genome RNAi screens in HER2 transformed BC cell lines revealed the IL6/JAK2/STAT3 axis as a master regulator pathway underlying the resistance phenotype against trastuzumab (Rodriguez-Barrueco et al., 2015). The identification of the master regulatory axis is then followed by the exploration of combination therapy that can inhibit both HER2 and IL6/JAK2/STAT3 axis. Interestingly, a combination consisting of trastuzumab and ruxolitinib – a JAK1/JAK2 inhibitor – demonstrated a synergistic cancer inhibition in mouse xenografts of HER2 transformed BC cell lines (Rodriguez-Barrueco et al., 2015).

However, certain technical and biological challenges must be mitigated preceding the fulfillment of the potential of multi-omics based precision medicine for cancer patients. First is the routine usage of omics technologies in a clinical setting that has not been widespread due to technical difficulties, reproducible analysis pipelines, and accessibility. With further technical and computational advancement, it may be possible to incorporate these multi-omics techniques in routine laboratory tests to facilitate the identification of personalized molecular-signature to determine the treatment-regimen of individual patients in the future. Second, collecting tissue samples for solid tumors at different time points through an invasive process such as biopsy from the chronologically distinct primary and secondary sites of a tumor, although it can be immensely beneficial to understand the molecular evolution of tumor and the mechanism of drug-resistance, it may not be practical in clinical settings. However, genomics studies on liquid biopsy - cancer cells/DNA fragments from a tumor that are circulating in the blood or other body fluids such as urine, may offer an alternative strategy to identify the dynamic molecular alterations associated with the evolution of tumor through the course of cancer progression (Mattox et al., 2019). The concept of liquid biopsy is gaining traction as an increasing number of studies are taking advantage of the availability of NGS technologies to analyze DNA samples in liquid biopsy samples to explore the tumor-specific genomic alterations. DNA analysis of liquid biopsy is based on the concept that clonal proliferation of tumor cells giving rise to tens of millions of cells carrying the identical mutated DNA, can serve as the template to be detected in blood samples (Mattox et al., 2019). The application of liquid biopsies at the various stages of cancer progression and therapeutic-regimen has proven to be beneficial. For instance, it has been shown that colon cancer patients with circulating tumor DNA following surgery are more likely to relapse, in contrast to the patients without circulating tumor DNA exhibiting less frequent relapse (Tie et al., 2016). In addition to genomics, the application of LC-MS/MS-based proteomics techniques is now being considered to explore the cancer-specific proteomic signatures in liquid biopsy samples. The proof of concept study utilizing proteomics technique on the urine samples collected from prostate cancer patients identified 133 differentially expressed protein-signatures (Kim et al., 2016).

The future of cancer research will likely be based on the concept of precision medicine and tracking of effective biomarkers that can detect the early stages of cancer thus allowing the clinicians to focus on preventive measures. Multi-omics driven systems biology approaches will be vital for discovering effective biomarkers and therapeutic strategies that are essential for precision medicine (Werner et al., 2014). In future, the systems biology approach may find its application in increasing the efficacy of targeted therapy by determining the optimal timing and dose and in formulating a strategy to bypass the emergence of resistance by suggesting a combination of therapies. Thus multi-omics driven systems biology may transform the effects of current targeted therapies into durable responses and subsequently improve the quality of life and provide a cure.

## Author Contributions

SC, GA, and MA conceptualized the manuscript. TD and SC retrieved and analyzed the data from various multi-omics sources to obtain the necessary statistics. TD, GA, and SC prepared the figures and tables. TD, GA, MA, and SC wrote the manuscript. All authors contributed to the article and approved the submitted version.

## Conflict of Interest

The authors declare that the research was conducted in the absence of any commercial or financial relationships that could be construed as a potential conflict of interest.

## Footnotes

1 https://portal.gdc.cancer.gov/

2 http://firebrowse.org/

3 https://www.cbioportal.org/

4 http://ngs.ym.edu.tw/driverdb/

5 https://tcpaportal.org/tcpa/

6 http://explorer.cancerregulome.org/

7 http://gepia.cancer-pku.cn/

8 https://xena.ucsc.edu/

9 http://ualcan.path.uab.edu/

10 http://maplab.imppc.org/wanderer/

11 https://cancer.sanger.ac.uk/cosmic

12 https://www.proteinatlas.org/

13 https://www.ncbi.nlm.nih.gov/geo/

14 https://www.ebi.ac.uk/pride/
